# Combined Effects of Turbulence and Different Predation Regimes on Zooplankton in Highly Colored Water—Implications for Environmental Change in Lakes

**DOI:** 10.1371/journal.pone.0111942

**Published:** 2014-11-06

**Authors:** Laura Härkönen, Zeynep Pekcan-Hekim, Noora Hellén, Anne Ojala, Jukka Horppila

**Affiliations:** Department of Environmental Sciences, University of Helsinki, Helsinki, Finland; CNRS, France

## Abstract

In aquatic ecosystems, predation is affected both by turbulence and visibility, but the combined effects are poorly known. Both factors are changing in lakes in the Northern Hemisphere; the average levels of turbulence are predicted to increase due to increasing wind activities, while water transparency is decreasing, e.g., due to variations in precipitation, and sediment resuspension. We explored experimentally how turbulence influenced the effects of planktivorous fish and invertebrate predators on zooplankton when it was combined with low visibility caused by high levels of water color. The study was conducted as a factorial design in 24 outdoor ponds, using the natural zooplankton community as a prey population. Perch and roach were used as vertebrate predators and *Chaoborus flavicans* larvae as invertebrate predators. In addition to calm conditions, the turbulent dissipation rate used in the experiments was 10^−6^ m^2^ s^−3^, and the water color was 140 mg Pt L^−1^. The results demonstrated that in a system dominated by invertebrates, predation pressure on cladocerans increased considerably under intermediate turbulence. Under calm conditions, chaoborids caused only a minor reduction in the crustacean biomass. The effect of fish predation on cladocerans was slightly reduced by turbulence, while predation on cyclopoids was strongly enhanced. Surprisingly, under turbulent conditions fish reduced cyclopoid biomass, whereas in calm water it increased in the presence of fish. We thus concluded that turbulence affects fish selectivity. The results suggested that in dystrophic invertebrate-dominated lakes, turbulence may severely affect the abundance of cladocerans. In fish-dominated dystrophic lakes, on the other hand, turbulence-induced changes in planktivory may considerably affect copepods instead of cladocerans. In lakes inhabited by both invertebrates and fish, the response of top-down regulation to turbulence resembles that in fish-dominated systems, due to intraguild predation. The changes in planktivorous predation induced by abiotic factors may possibly cascade to primary producers.

## Introduction

One of the main shortcomings in understanding the response of aquatic ecosystems to disturbances is the lack of a framework blending together physics and biology [1]. This also holds in climate change studies in which most scenarios on the effects on aquatic ecosystems have focused on rising water temperature, variations in external nutrient loading, and resulting changes in nutrient concentrations [2], [3], [4], while one of the most important physical factors, water turbulence, and its effects on biological interactions have been ignored. Changes in mixing depth have been included in predictive models [5], but changes in water column turbulence, which is the irregular, diffusive, dissipative flow of water without any preferred velocity direction [6], have mostly been neglected. Turbulence affects aquatic ecosystems, e.g. via bottom-up regulation, because it influences nutrient cycling and light environment through sediment resuspension and resulting water turbidity. Such effects have been widely studied [7], [8] and have also been included in climate change scenarios [3].

Turbulence also affects predator-prey interactions [9], [10], which is noteworthy, because predation largely regulates lacustrine population dynamics [11]. Predation affects the density, biomass, size structure, as well as behavior of prey populations. The effects of predation are again dependent on the prevailing predator. For instance, predation pressure by juvenile and adult stages of planktivorous fish usually results in a zooplankton community dominated by small species [12], [13], whereas predation by invertebrates often leads to dominance by large-bodied zooplankton [14], [15]. Changes in the predation regime thus strongly affect zooplankton communities, and changes in zooplankton can cascade down the food web to primary producers [16]. The strength of zooplanktivory is affected by numerous environmental factors, such as availability of refuges for zooplankton against predation, light intensity, and water turbulence [9], [17], [18], [19]. The importance of each factor is dependent on the characteristics of the predator and the prey.

Small-scale turbulence enhances planktonic ingestion rates, due to increased encounter rates between predators and prey [9], [20], [21]. Due to differences in swimming speed, the effect of turbulence is size-dependent, and larger organisms, such as fish more than a few centimeters in body length, are often assumed to be unaffected by turbulence [22]. On the other hand, some studies have controversially shown that turbulence has no positive effects on invertebrate predators and larval fish, while the feeding of larger fish may be positively affected [23], [24], [25]. It was suggested that the positive effects of turbulence only operate under food conditions below the saturation level [20], [26]. However, little is known of the effect of turbulence on prey selection of predators when the prey population is versatile. If turbulence affected selective predation, the subsequent effects on the zooplankton community structure could occur even if prey densities were optimal.

Turbulence interacts with other environmental parameters, which complicates the studies. The effects of turbulence on predation may vary with visibility, since low visibility reduces the reactive distance of predators, while at the same time turbulence may increase the number of prey items entering predators' reactive volume [25], [27]. This is crucial, because most fish are visual feeders, whereas many invertebrate predators are tactile predators detecting their prey by mechano- and chemoreception [28]. At low visibility, invertebrate predators may thus predominate over planktivorous fish [29], [30].

In all, the relationship between turbulence and predation is more complicated than generally assumed [31]. For instance, little is known of the effects of turbulence in aquatic ecosystems with low visibility, such as brown-water lakes, which are abundant in the Boreal Zone [32]. Moreover, both turbulence and visibility in lakes are changing on a large scale. Climate models predict increasing wind speeds in Northern Europe, with consequences for the turbulence levels of aquatic ecosystems [33], [34]. The reductions predicted in the water level of many lakes will also affect the turbulence conditions [35], [36]. At the same time, water transparency in numerous lakes is decreasing, due to increasing loads of suspended solids and sediment resuspension, and through increased loading of dissolved organic matter (DOM), all of which lead to increased water turbidity and/or water brownification [2], [37], [38].

The present experimental study explores the combined effects of turbulence and low visibility caused by high levels of water color on predation by planktivorous fish and invertebrate predators on crustacean zooplankton. Since both intermediate turbulence and low visibility should be more beneficial for tactile invertebrate predators than for fish [22], [28], we hypothesized that the effects of predation on zooplanktonic prey populations should be considerably stronger in a turbulent, invertebrate-dominated brown-water system than in a comparable fish-dominated system. The results contribute to an understanding of the impacts of the ongoing environmental change on zooplankton communities of brown-water lakes via the effects on predation. The study focuses on crustacean zooplankton, but data on rotifers are also presented.

## Materials and Methods

### Experimental setup

**Figure 1:**
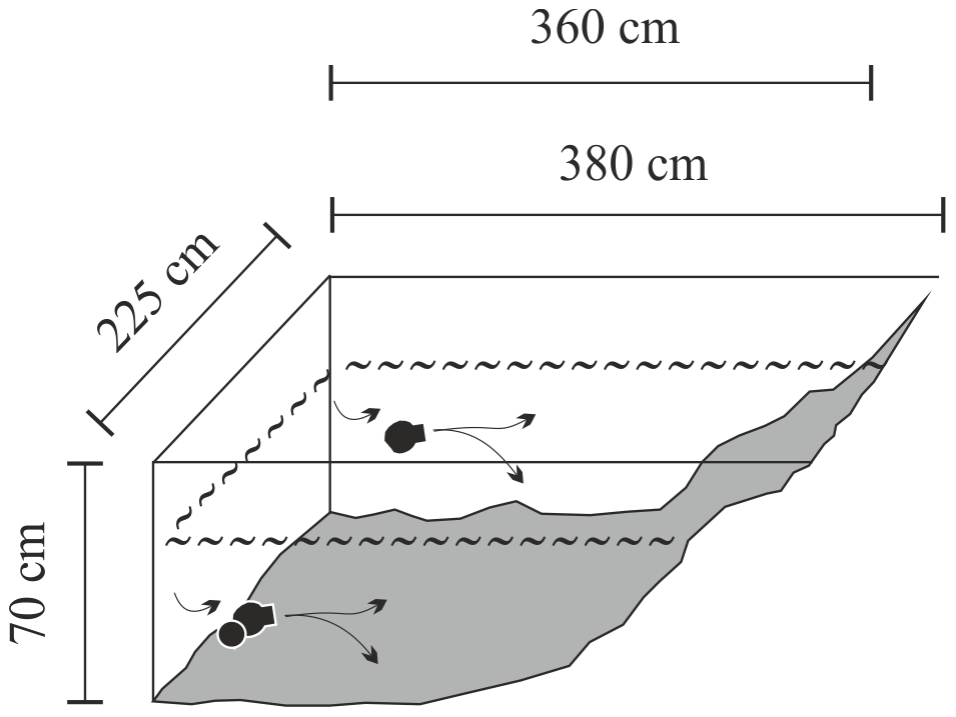
A schematic presentation of a single experimental pond with dimensions. The wavy dashed line indicates the water table (the maximum depth of the ponds was 60 cm with an average depth of 40 cm). Submersible pumps were placed at middepth; the single arrows represent water inlets and the split arrows the water outlets of the pumps.

The experiments were conducted as a 2×2×2 factorial design, including four different predation regimes: no predation (CNTRL), invertebrate predation (I), fish predation (F), invertebrate + fish predation (IF), two different turbulence conditions: no induced turbulence (CALM), intermediate turbulence (TURB), and three replicates for each combination of turbulence and predators. The experiments were conducted between July 23 and August 31 2012 in 24 experimental outdoor ponds (coordinates 61°12'N, 25°8'E, each 8.1 m^2^ in surface area with volume of 3200 L) situated in the Evo district in southern Finland. The ponds were rectangular in shape, with sand-gravel bottoms with 0.5–1-cm layer of organic debris and no vegetation (Fig. 1). The maximum depth of the ponds was 60 cm and the average depth 40 cm. In such shallow ponds, zooplankton could not escape turbulence by downward migrations [39]. After being drained for 2 weeks, 10 d prior to the experiments, the ponds were filled with water filtered through a 50-µm net from the nearby humic Lake Majajärvi (61°12'N 19°8'E). The water color was 140 mg Pt L^−1^ (measured after filtration). Lake Majajärvi is a typical small forest lake with a surface area of 3.8 ha, a mean depth of 4.6 m, and a maximum depth of 12 m. It has abundant planktivorous fish stocks (Eurasian perch *Perca fluviatilis* L. and roach *Rutilus rutilus* (L.) and is inhabited by planktivorous phantom midge larvae (*Chaoborus flavicans* Meigen) [40], [41].

### Turbulence generation and measurement

Turbulence was generated by computer-controlled submersible pumps (Tunze Turbelle Nanostream 6055; Tunze Aquarientechnik GmbH, Penzberg, Germany) to create the desired magnitude of turbulence. The pumps had a flow rate of 1000 L h^−1^ and an output diameter of 4 cm. In each pond with turbulence, two pumps were placed on opposite sides (Fig. 1). Pumps disturb the behavior of fish less than do oscillating grids and pump-generated turbulence has been used in previous studies [25], [42], [43], [44].

Turbulence was measured, using an acoustic Doppler velocimeter (ADV, 10-MHz ADVField; Sontek/YSI, San Diego, CA, USA). To determine the turbulence, a 25-Hz measurement for a period of 2 min was conducted from the middle of the water column at nine points around the ponds. From the data provided by a HorizonADV 1.20 (Sontek/YSI) the root-mean-square (*RMS*) velocities (cm s^−1^) were calculated:
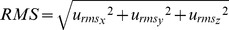
(1)where
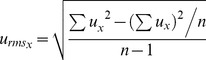
(2)which is the fluctuation of the flow for Cartesian vector x; *u_x_* is the velocity of vector x, and *n* is the number of samples in a 2-min measurement. The *RMS* velocities were expressed as averages for the whole pond. The energy dissipation rate, *?* (m^2^ s^−3^), which describes the rate at which turbulent energy decays over time, was calculated for the average *RMS* velocities (m s^−1^) [45]

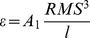
(3)where *A*_1_ is a nondimensional constant of order 1 [46], [47] and *l* is the water depth (m) that describes the size of the largest vortices. The Reynolds (*Re*) numbers (the ratio of inertial forces to viscous forces) were calculated [48]:
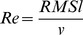
(4)where *l* is the water depth (m) and *v* is the kinematic viscosity for water (10^−6^ m^2^ s^−1^). Such equations were used to calculate *?* and *Re*, due to the simple turbulence vs. no turbulence arrangement applied in the experiments.

The average *RMS* velocity for the turbulent ponds was adjusted to 1.4 cm s^−1^ (±0.09 cm s^−1^) with a corresponding *?* value of 5.6×10^−6^ m^2^ s^−3^ (±1.1×10^−6^ m^2^ s^−3^) and an *Re* of 7438 (±562). The *RMS* velocity within each experimental unit varied between 0.7 and 2.4 cm s^−1^. The background turbulent *RMS* velocity for the ponds with no added turbulence was 0.3 cm s^−1^ on average (±0.1 cm s^−1^) with a corresponding *?* value of 4.6×10^−8^ m^2^ s^−3^ and an *Re* of 1770. During calm conditions in lakes, the dissipation rate in the surface mixed layer often varies between 10^−9^ and 10^−8^ m^2^ s^−3^, and may rise to a level between 10^−6^ and 10^−5^ m^2^ s^−3^ during wind forcing [49], [50]. The magnitude of turbulence in the ponds was thus adjusted to an intermediate level often occurring in lakes during mixing and which, unlike high turbulence, does not physically harm most crustacean zooplankton species [36].

### Predators and prey

A mixture of the natural zooplankton community of Lake Majajärvi was collected by horizontal net hauls, using a 153-µm plankton net, the sampled volume of water representing the combined volume of all 24 ponds. Subsequently, equal aliquots of zooplankton were added to each pond on July 17. Chaoborids were removed from the samples, but smaller crustacean predators such as cyclopoid copepods and predatory cladocerans were included in natural densities. The zooplankton were allowed to acclimatize and develop for 7 d before the experiment was initiated. Finnish law stipulates that no permits are required for field sampling of plankton.

Phantom midge larvae (*C*. *flavicans*), used as invertebrate predators, were collected from Lake Majajärvi by net hauls. Chaoborids are an important prey item for fish, but at the same time they are considered as one of the most abundant and important invertebrate predators in freshwater communities [51], [52]. In numerous lakes, chaoborids and fish co-occur at high densities [52], [53]. Each experimental pond with an invertebrate predator treatment received 960 larvae, leading to an initial density of 0.3 ind. L^−1^ (119 ind. m^−2^), corresponding to moderate densities found in many lakes [40], [54]. Such moderate density was chosen to aid the detection of the possible effects of turbulence. With moderate densities of chaoborids, both strong and weak effects on prey populations have been detected, indicating that environmental variables may regulate their predation efficiency [54], [55]. At high *Chaoborus* densities (>1000 ind. m^−2^), the effects on prey populations have mostly been strong [52], [56], [57]. The length of the larvae used in the experiments was 8.5±1.9 mm, and they represented instars III and IV. Each week 80 larvae were added to the I and IF treatments to compensate for the emergence of larvae, the number of added individuals corresponding to the proportion (9%) of pupae in the *C. flavicans* community of Lake Majajärvi in late July and August [58].

Perch (total length 8.0±2.3 cm) and roach (total length 8.0±2.1 cm), common in boreal humic lakes, were used in the experiments as vertebrate predators. Three individuals of each species were introduced to the F and IF ponds, resulting in a 34-kg ha^-1^ fish biomass, a natural level of fish biomass in numerous humic lakes [41], [54]. The fish were collected from Lake Majajärvi by trap-netting (mesh size 1.5 cm). The fish captured were transported a 300-m distance from the lake to the ponds in 40-L buckets containing water taken from Lake Majajärvi. They were left to acclimatize for 1 week in a pond which was filled with Lake Majajärvi water, and excluded from the experiments. The fish were captured from the acclimatization pond by hauling, measured for length, and placed in the experimental ponds 1 d after the *Chaoborus* larvae, allowing the chaoborids to acclimatize and avoid artificially high predation losses to fish.

The fish and *Chaoborus* larvae were collected from Lake Majajärvi with permission of the Finnish National Board of Forestry (Metsähallitus, Permit Number: 31875). No endangered species were involved in the study. Ethical concerns on the care and use of experimental animals were followed under permission approved by the Finnish Animal Welfare Commission (Permit Number: STH188A). No vertebrates were sacrificed in the study; after the study period, the experimental fish were captured from the experimental ponds by trap netting and released back to Lake Majajärvi.

### Sampling and analyses

Seven days after the zooplankton mixture was added to the ponds, zooplankton samples were taken with a tube sampler (5.4-cm diameter, 50-cm length) from five random places around each pond (total sample volume 6 L per pond) to determine the initial zooplankton community structure. The tube was rapidly passed through the water column, allowing water to enter it, and immediately sealed and lifted up from the water. The bulk samples were concentrated with a 50-µm plankton net and preserved in 4% formaldehyde. The turbulence was initiated after taking these zero samples. Four days later, the zooplankton were sampled again, after which the *C*. *flavicans* larvae and fish 1 d later were added. The ponds were sampled at 4-d intervals for 6 weeks. The zooplankton samples were analyzed by inverted microscopy (Olympus CK40, 125x magnification; Olympus Corporation, Tokyo, Japan) and identified to species or genus level. From each crustacean taxon, 30 individuals were measured. *Daphnia* sp. were measured from the center of the eye to the base of the tail and other species from the anterior edge to the posterior edge of the carapace. The zooplankton biomasses were calculated from individual lengths, using length-weight regressions [59], [60], [61], [62].

During each sampling, water temperature, dissolved oxygen (DO), and pH were determined at the middepth from each pond (YSI 6600V2 sonde (YSI Inc., Yellow Springs, OH, USA) and the light intensity was determined with an LI-192SA quantum sensor (LI-COR Biosciences, Lincoln, NE, USA) equipped with an LI-1400 datalogger. Total phosphorus (TP) and total nitrogen (TN) samples were taken with a tube sampler and analyzed, using the method of Koroleff [63] with a Lachat autoanalyzer (QuickChem Series 8000; Lachat Instruments (Hach Company), Loveland, CO, USA). Chlorophyll *a* (Chl *a*) samples were taken, filtered through Whatman GF/C filters, and analyzed spectrophotometrically (Shimadzu UV-260, UV-Visible Recording Spectrophotometer; Shimadzu Corporation, Tokyo, Japan) after extraction with ethanol [64].

### Statistical analysis

The between treatment-differences in the initial zooplankton biomass were studied by analyzing the results of the first sampling day with analysis of variance (ANOVA) (ln(x+1)-transformed data). The groups analyzed included crustaceans, cladocerans, *Bosmina* spp., daphnids, chydorids, *Polyphemus pediculus* (L.), copepods, cyclopoids, calanoids, and rotifers. The effects of the various treatments on the biomass of various zooplankton taxa were studied with analysis of variance for repeated measurements (ANOVAR), which accounts for the temporal autocorrelation between sequential samples (ln(x+1)-transformed data). Pairwise comparisons between treatments were conducted with Bonferroni t-tests. Additionally, to study the effects of turbulence on the size selectivity of predation, the proportions of the various size classes in the biomass of the crustacean zooplankton (all species combined) were compared between treatments, including predators (arcsine √*x* – transformed data). To determine the possible bottom-up effects of turbulence on zooplankton via effects on the phytoplankton biomass, the between-treatment differences in Chl *a* were analyzed with ANOVAR.

## Results

### Physicochemical water quality

For most of the study period, the water temperature fluctuated between 18 and 21°C. In late July, the temperature temporarily reached 23°C. The between-treatment differences were <0.5°C (Table 1). Depending on the weather, the light intensity 5 cm below the surface fluctuated between 100 and 600 µmol m^−2^ s^−1^, the average value being 240 µmol m^−2^ s^−1^. In the bottom layers, the average light intensity was 63 µmol m^−2^ s^−1^. No differences between treatments were detected; the light extinction coefficient was on average 4.8 m^−1^ in the CALM ponds and 4.9 m^−1^ in the TURB ponds. The concentration of DO varied between 8 and 9 mg L^−1^ and water pH between 6.8 and 6.9. The average concentration of total nutrients varied between 16 and 21 µg TP L^−1^ and 800 and 850 µg TN L^−1^, and Chl *a* concentration between 13 and 19 µg L^−1^ (Table 1). The chlorophyll *a* concentration did not differ between treatments (ANOVAR, F_7,16_  = 1.241, p = 0.244).

**Table 1:** Average values (± standard deviation) of water temperature, dissolved oxygen (DO), pH, total phosphorus (TP) and nitrogen (TN), and chlorophyll *a* (Chl *a*) in the various treatments during the study period in calm (CALM) and turbulent (TURB) water.

Treatment	Temp (°C)	DO (mg L^−1^)	pH	TP (µg L^−1^)	TN (µg L^−1^)	Chl *a* (µg L^−1^)
CALM-CNTRL	18.7±2.1	8.3±0.4	6.9±0.2	19±6	845±84	16.1±9.1
CALM-I	18.7±2.1	8.3±0.5	6.9±0.2	16±4	796±75	14.9±8.0
CALM-F	18.6±2.1	8.4±0.4	6.9±0.1	18±5	830±88	18.3±13.7
CALM-IF	18.5±2.0	8.3±0.5	6.9±0.1	20±11	859±106	18.5±11.4
TURB-CNTRL	18.8±2.1	8.7±0.3	6.9±0.1	19±5	834±78	13.3±6.4
TURB-I	18.9±2.1	8.5±0.3	6.9±0.1	18±6	821±98	15.6±7.2
TURB-F	18.7±2.1	8.6±0.3	6.8±0.1	20±9	849±100	17.0±7.9
TURB-IF	18.7±2.1	8.7±0.3	6.8±0.1	19±6	829±72	16.3±7.7

The predator treatments were: CNTRL  =  control, no added predators, I =  invertebrate predators (*Chaoborus flavicans*), F =  fish, and IF  =  both invertebrates and fish as predators.

### Crustacean zooplankton

The crustacean zooplankton were dominated by cladocerans, *Bosmina* spp. being the most abundant taxa (mainly *B*. *longirostis* O. F. Müller) (Table 2). Their average density varied between 12.1 and 31.5 ind. L^−1^, being lowest in CALM-IF and highest in CALM-CNTRL. The density of *Polyphemus pediculus* exceeded 10 ind. L^−1^ in CALM-CNTRL and CALM-I. The density of daphnids and chydorids was <1 ind. L^−1^ in all treatments (Table 2). *Ceriodaphnia quadrangula* O. F. Müller attained densities >1 ind. L^−1^ in all the treatments (Table 2). Cyclopoid copepods were dominated by *Mesocyclops* sp. (0.3–1.1 ind. L^−1^), with the highest density in CALM-F and lowest in TURB-I and TURB-F. Calanoid copepods were dominated by *Eudiaptomus gracilis* G. O. Sars (0.2–0.6 ind. L^−1^), with the highest densities in the CNTRL treatments.

**Table 2:** Average density ± standard deviation (ind. L^−1^) of crustacean zooplankton in the various treatments throughout the study period in calm (CALM) and turbulent (TURB) water.

Zooplankton Taxa	CALM-CNTRL	CALM-I	CALM-F	CALM-IF	TURB-CNTRL	TURB-I	TURB-F	TURB-IF
*Alona* sp.		0.03±0.14		0.01±0.06			0.68±0.06	
*Alonella* sp.	0.03±0.15		0.04±0.19		0.03±0.13	0.06±0.28	0.03±0.14	0.04±0.21
*Bosmina* spp.	31.48±39.00	29.30±38.39	23.30±28.50	12.06±11.98	22.38±31.31	15.03±22.11	15.47±23.68	16.82±23.92
*Ceriodaphnia* sp.	1.62±2.74	4.80±8.08	3.02±3.30	1.11±1.71	1.16±2.60	1.18±2.08	1.04±2.21	1.51±2.65
*Chydorus* sp.					0.01±0.06			
*Daphnia* sp.	0.29±1.21	0.23±0.92	0.05±0.25	0.36±0.09	0.30±0.96	0.27±1.07	0.18±0.79	0.54±1.80
*Polyphemus pediculus*	14.56±13.66	12.51±10.07	0.34±0.85	0.47±0.94	5.23±6.60	1.47±2.65	0.72±2.52	0.73±2.11
*Scapholeberis* sp.	1.74±3.50	0.87±1.42	0.67±1.12	0.31±0.55	0.71±1.73	0.16±0.42	0.44±0.79	0.26±0.64
Cladocera embryo	19.38±22.99	16.40±15.05	9.28±14.60	5.12±10.09	14.67±27.30	6.98±13.74	5.46±9.04	5.89±9.69
								
*Eudiaptomus gracilis*	0.71±1.27	0.36±0.52	0.23±0.68	0.18±0.51	0.57±1.06	0.33±1.03	0.11±0.61	0.24±0.64
Calanoida copepod.	0.02±0.12	0.16±0.54	0.13±0.37	0.08±0.37	0.04±0.24	0.10±0.32	0.04±0.24	0.16±0.41
Calanoida nauplii	0.11±0.35	0.16±0.38	0.38±0.89	0.08±0.27	0.19±0.58	0.13±0.54		0.06±0.25
								
*Cyclops* sp.				0.02±0.12	0.06±0.25			0.03±0.13
*Mesocyclops* sp.	0.36±0.57	0.67±1.33	1.12±1.62	0.64±0.93	0.50±1.50	0.31±0.91	0.31±1.03	0.84±1.52
Cyclopoida copepod.	1.79±3.67	1.48±2.14	3.71±3.67	2.30±2.62	1.16±1.89	1.07±1.82	1.61±2.09	2.17±2.73
Cyclopoida nauplii	15.70±18.72	13.70±13.05	28.24±21.80	20.59±20.25	10.04±11.10	10.69±10.73	13.41±12.84	16.86±15.49

The predator treatments were: CNTRL  =  control, no added predators, I =  invertebrate predators (*Chaoborus flavicans*), F =  fish, and IF  =  both invertebrates and fish as predators. The empty cells indicate that there were no individuals detected during the study period.

There were no between-treatment differences in the initial zooplankton biomass in any of the zooplankton groups studied (one-way ANOVA, p>0.1 for all taxa). Throughout the study period, all the taxa analyzed were affected by the treatment (ANOVAR) (F_7,2080_  = 14.949, p<0.001), sampling day (F_9,2080_  = 23.346, p<0.001), as well as the treatment×day interaction (F_63,2080_  = 1.335, p<0.05) in terms of biomass.

**Figure 2:**
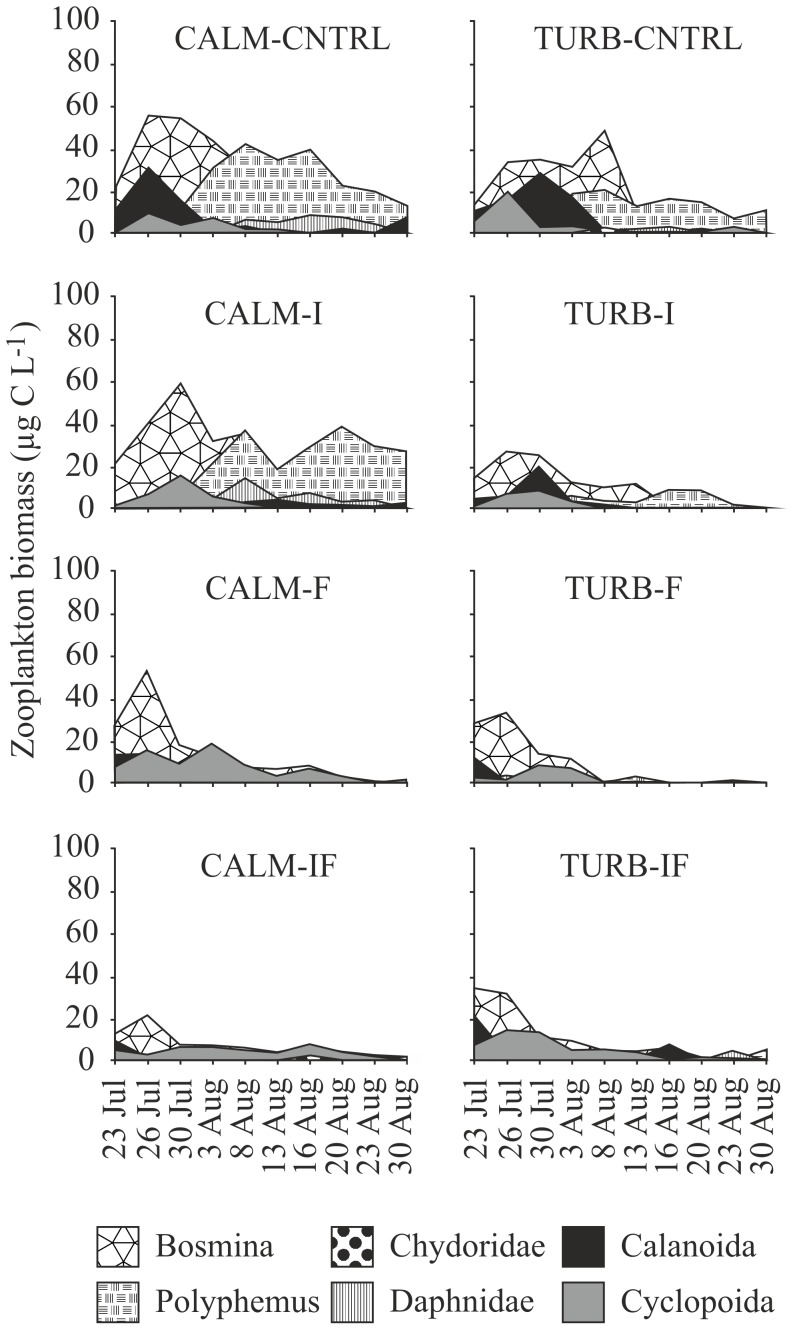
Development of crustacean zooplankton biomass in the various treatments during the study period in calm (CALM) and turbulent (TURB) water. Turbulence was initiated after the sampling on July 23, and the predators were added after the sampling on July 26. The predator treatments were: CNTRL  =  control, no added predators, I =  invertebrate predators (*Chaoborus flavicans*), F =  fish, and IF  =  both invertebrates and fish as predators.

In pairwise comparisons, the biomass of cladocerans was lower in TURB-CNTRL than in CALM-CNTRL (Fig. 2, Table 3a). In both of these treatments, *Bosmina* spp. dominated the first half of the experiment, reaching 30 µg C L^−1^ biomass. The biomass of cyclopoid and calanoid copepods was mostly <10 µg C L^−1^ and was not affected by turbulence alone (Table 3a). The lower biomass of cladocerans in TURB-CNTRL was due to the negative effect of turbulence on *P*. *pediculus* (Table 3a). In both CNTRL treatments, *P*. *pediculus* dominated the crustacean community in the latter half of the experiment, but the biomass was lower in turbulent (max. 20 µg C L^−1^) than in calm water (max. 38 µg C L^−1^) (Fig. 2). In the biomass of cladoceran embryos or copepod nauplii, there were no differences between treatments (ANOVAR, Bonferroni t-tests, p>0.05). Additionally, no differences were observed between treatments either in the adult-embryo ratio of cladocerans (ANOVAR, Bonferroni t-tests, p>0.05) or in the adult-nauplii ratio of copepods (ANOVAR, Bonferroni t-tests, p>0.05).

**Table 3:** Pairwise between-treatment comparisons (ANOVAR, Bonferroni t-tests) for the differences in crustacean zooplankton biomass in calm (CALM) and turbulent (TURB) water.

Treatment	Cladocera	Daphnidae	*Bosmina* spp.	Chydoridae	*Polyphemus*	Copepoda	Cyclopoida	Calanoida
**(a)** Effects of turbulence alone								
CALM-CNTRL vs. TURB CNTRL	**	-	-	-	**	-	-	-
								
**(b)** Calm control vs. calm water with predators								
CALM-CNTRL vs. CALM-I	-	-	-	-	-	-	-	-
CALM-CNTRL vs. CALM-F	**	-	-	-	**	-	**	-
CALM-CNTRL vs. CALM-IF	**	-	**	-	**	-	-	-
								
**(c)** Turbulent control vs. turbulent water with predators								
TURB-CNTRL vs. TURB-I	**	-	*	-	**	-	-	-
TURB-CNTRL vs. TURB-F	**	-	*	-	**	-	-	*
TURB-CNTRL vs. TURB-IF	**	-	-	-	**	-	*	-
								
**(d)** Different predators in calm water								
CALM-I vs. CALM-F	**	-	-	-	**	-	**	-
CALM-I vs. CALM-IF	-	**	**	-	**	-	-	-
CALM-F vs. CALM-IF	-	-	-	-	-	-	-	-
								
**(e)** Different predators in turbulent water								
TURB-I vs. TURB-F	-	-	-	-	**	-	*	-
TURB-I vs. TURB-IF	-	-	-	-	**	**	**	-
TURB-F vs. TURB-IF	-	-	-	-	-	*	*	-
								
**(f)** Calm vs. turbulent water with different predators								
CALM-I vs. TURB-I	*	**	**	-	**	-	-	-
CALM-F vs. TURB-F	-	-	*	-	-	**	**	-
CALM-IF vs. TURB-IF	-	-	-	-	-	-	-	-

Predator treatments were: CNTRL  =  control, no added predators, I =  invertebrate predators (*Chaoborus flavicans*), F =  fish, and IF  =  both invertebrates and fish as predators. (** p<0.01, * p<0.05, - no significant difference).

In calm water, chaoborid predation did not affect the biomass of any zooplankton group (Fig. 2, Table 3b). Bosminids predominated in CALM-I during the first weeks of the experiment, reaching a biomass of 58 µg C L^−1^ and were replaced by *P*. *pediculus* and daphnids in August. With fish predation in calm water, the biomass of *P*. *pediculus* was lower, and the biomass of cyclopoid copepods (max. 15 µg C L^−1^) higher than in CALM-CNTRL, but in other taxa no differences were observed (Fig. 2, Table 3b). When both fish and chaoborids were present in calm water, the biomass of *P. pediculus* and *Bosmina* spp. was lower than in CALM-CNTRL. In turbulent water, with all predator regimes, the biomass of *P*. *pediculus* was significantly lower than in TURB-CNTRL (Table 3c). Under turbulence, the biomass of bosminids was reduced by chaoborids and by fish, but not by their combined predation (Fig. 2, Table 3c). The biomass of cyclopoids was elevated under turbulence when both chaoborids and fish were present (Table 3c).

In comparing the various predation regimes in calm water, the biomass of *P. pediculus* was lower and that of cyclopoids higher in the presence of fish than in the presence of chaoborids (Fig. 2, Table 3d). With combined predation of chaoborids and fish, daphnids and bosminids were depressed, compared with chaoborid predation. No differences were observed in the effects of combined predation and fish predation (Table 3d). Among the various predator regimes in turbulent water, the main differences were in cyclopoids and *P*. *pediculus*. When both fish and chaoborids were present, the biomass of cyclopoids was higher than in single-predator treatments, while the biomass of *Polyphemus* was lowered in both treatments including fish (Table 3e).

Comparisons of fixed predation regimes in the CALM and TURB treatments showed that with invertebrate predators, the effects of turbulence were strongest on cladocerans, whereas in a fish-dominated system the effect on cladocerans was weaker, but copepods especially were affected. When chaoborids were the predators, all cladocerans except chydorids showed lower biomasses in turbulent than in calm water (Fig. 2, Table 3f). In contemplating the total average biomasses during the study period, the average biomass of cladocerans in CALM-I was 96% and the biomass of cyclopoids 123% of that in CALM-CNTRL. Under turbulent conditions in TURB-I, the biomasses compared with TURB-CNTRL (thus excluding the direct effect of turbulence on zooplankton) were 50% for cladocerans and 61% for cyclopoids. In treatments including fish, the biomass of bosminids and cyclopoids was lower under turbulence than in calm water (Fig. 2). In CALM-F cladoceran biomass was reduced to 34%, while the biomass of cyclopoids was elevated to 250% of that in CALM-CNTRL. Fish in turbulence, on the other hand, reduced cladoceran biomass to 38% and the biomass of cyclopoids to 71% of that under turbulent predator-free conditions. With combined predation (IF), no differences were detected between turbulent and calm water.

### Rotifers

**Figure 3:**
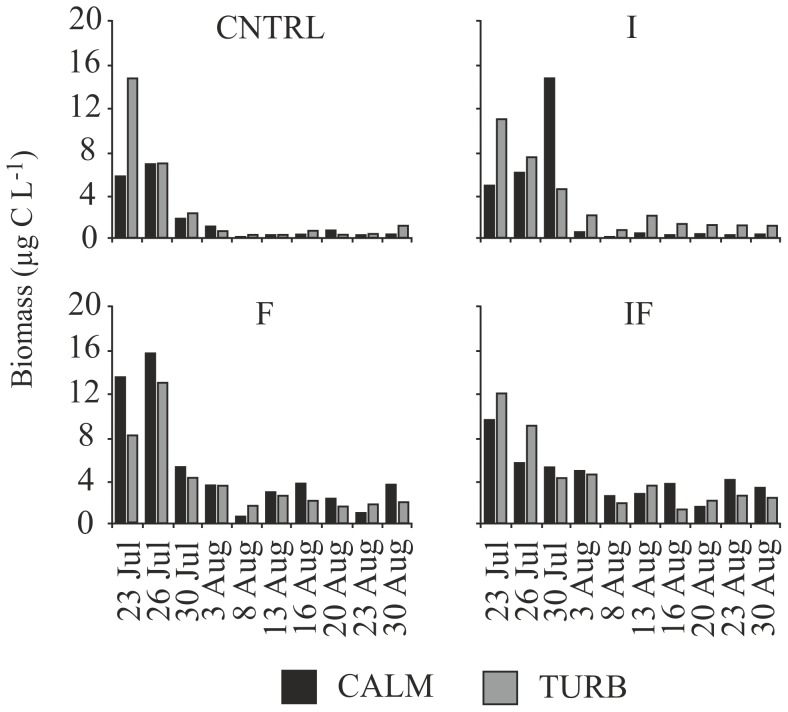
Development of rotifer biomass in the various treatments during the study period in calm (CALM) and turbulent (TURB) water. The predator treatments were: CNTRL  =  control, no added predators, I =  invertebrate predators (*Chaoborus flavicans*), F =  fish, and IF  =  both invertebrates and fish as predators.

The biomass of rotifers decreased in all treatments. During the first 2 weeks of the experiment, the biomass fluctuated between 4 and 16 µg C L^−1^ but dropped to <4 µg C L^−1^ in August in all treatments. However, differences were also detected in between-treatment comparisons. In CALM ponds, treatments including fish showed higher biomass of rotifers than CNTRL and *Chaoborus* treatments (ANOVAR, Bonferroni t-tests, p<0.05) (Fig. 3). In turbulent ponds on the other hand, treatments including fish had significantly higher rotifer biomasses than CNTRL treatments (ANOVAR, Bonferroni t-tests, p<0.05), but not *Chaoborus* treatments (ANOVAR, Bonferroni t-tests, p>0.05). Turbulence alone did not affect rotifer biomass, since the CNTRL treatments did not differ (ANOVAR, Bonferroni t-tests, p>0.05). When the CALM treatments were compared with the TURB treatments with fixed predator regimes, no differences in rotifer biomasses were observed (ANOVAR, Bonferroni t-tests, p>0.05). In all treatments, the rotiferan community was dominated by *Synchaeta* spp. in the first half of the experiment, *Keratella* sp., *Polyarthra* spp., and *Conochilus* spp. being the next most abundant taxa. Towards the end of the experiment, the proportions of *Ascomorpha* sp., and *Chromogaster* sp. increased.

### Size distribution of crustacean zooplankton

Turbulence alone did not affect the average size of the taxa studied, since no differences between the CALM- and TURB-CNTRL treatments were observed in any of the taxa (ANOVA, Bonferroni t-tests, p>0.05). The average individual size of bosminids was between 200 and 300 µm, and daphnids between and 400 and 500 µm in all the treatments. The average individual size of *P*. *pediculus* was 500–700 µm. In calanoid copepods, the average size was 950–1250 µm. In cyclopoid copepods, the average size fluctuated between 450 and 550 µm.

**Figure 4:**
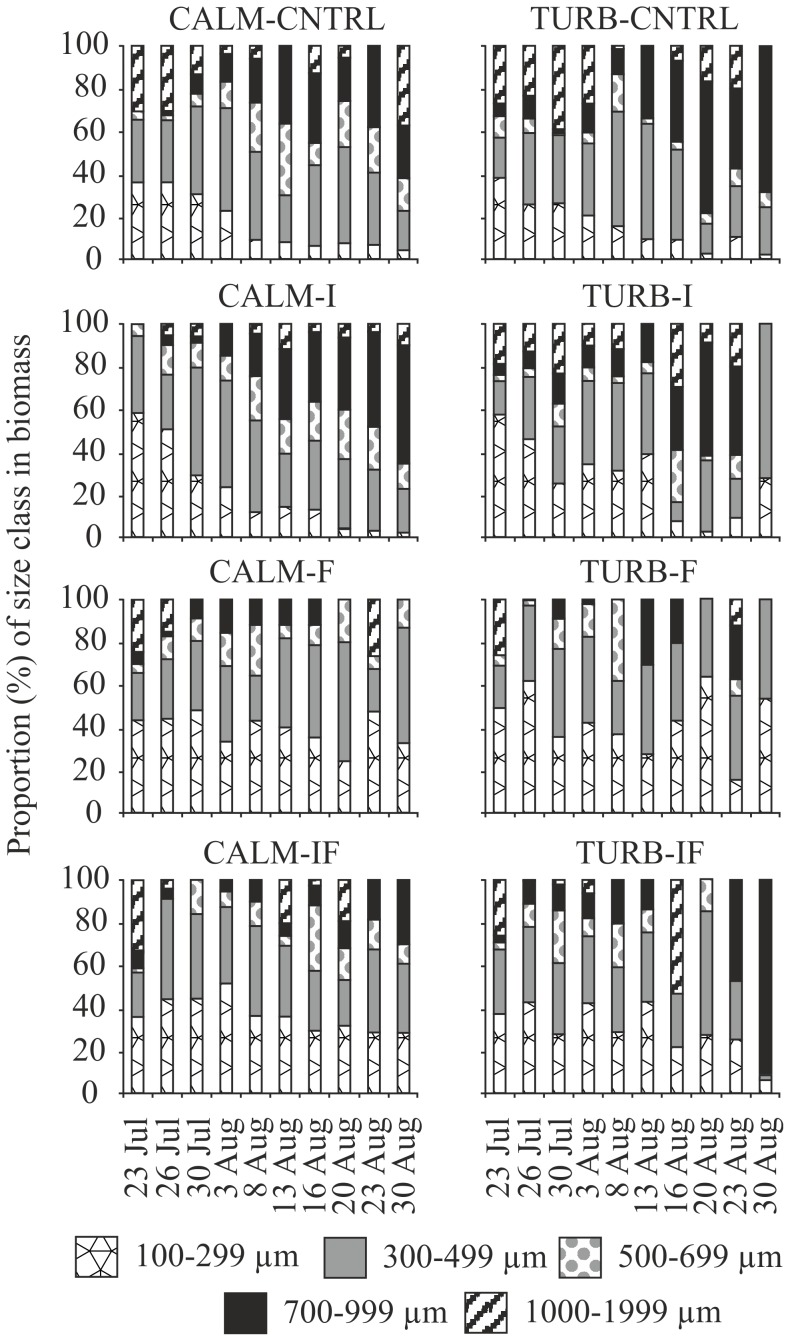
Proportion of different size classes of crustacean zooplankton biomass in the various treatments during the study period in calm (CALM) and turbulent (TURB) water. The predator treatments were: CNTRL  =  control, no added predators, I =  invertebrate predators (*Chaoborus flavicans*), F =  fish, and IF  =  both invertebrates and fish as predators.

When the various taxa were combined into size classes and their development in the course of the experiment was studied, clear trends were revealed in some of the treatments. At the beginning of the experiment, crustacean zooplankton in all the treatments were dominated by the small size classes 100–299 µm and 300–499 µm, which together formed >70% of the total biomass of crustaceans (Fig. 4). Thereafter, in both CNTRL treatments and in CALM-I, the proportion of the small size classes decreased steeply, while that of larger zooplankton increased (Fig. 4). In late August, the proportion of size classes 100–299 µm and 300–499 µm together was <30% in all these three treatments. In TURB-I, the decrease in small size classes was less clear, although towards the end of the study larger (>500 µm) size classes tended to predominate. In CALM-F and TURB-F, no trend in the size distribution of zooplankton was observed during the study period, but the proportions of the various size classes remained similar throughout the experiment (Fig. 4). In both of these treatments, the proportion of size classes 100–299 µm and 300–499 µm together remained above 60% throughout the experiment. In CALM-IF and TURB-IF, the proportions of the various size classes were similar to those in the F treatments, showing no trends during the experiment (Fig. 4).

When the biomass of the various size classes was compared between the CALM and TURB treatments with different predators, the only significant difference was between the I treatments in the proportion of size classes 500–699 µm and 700–999 µm (Table 4a). No differences in the smallest (100–499 µm) and largest (>1000 µm) size classes were detected. When the various predation regimes within CALM and TURB treatments were compared, the proportion of the size class 700–999 µm was higher in CALM-I than in CALM-F (Table 4b). When turbulence was present, the proportions of size classes 700–999 µm and >1000 µm were higher in TURB-CNTRL than in TURB-I (Table 4c).

**Table 4:** Pairwise comparisons for the proportion of biomass in different size classes (µm) of crustacean zooplankton in predator treatments in calm (CALM) and turbulent (TURB) water (ANOVAR, Bonferroni t-tests).

Treatment	100–299	300–499	500–699	700–999	>1000
**(a)** Calm vs. turbulent water with different predators					
CALM-CNTRL vs. TURB-CNTRL	-	-	-	-	-
CALM-I vs. TURB-I	-	-	**	*	-
CALM-F vs. TURB-F	-	-	-	-	-
CALM-IF vs. TURB-IF	-	-	-	-	-
					
**(b)** Different predators in calm water					
CALM-CNTRL vs. CALM-I	-	-	-	-	-
CALM-CNTRL vs. CALM-F	-	-	-	-	-
CALM-CNTRL vs. CALM-IF	-	-	-	-	-
CALM-I vs. CALM-F	-	-	-	*	-
CALM-I vs. CALM-IF	-	-	-	-	-
CALM-F vs. CALM-IF	-	-	-	-	-
					
**(c)** Different predators in turbulent water					
TURB-CNTRL vs. TURB-I	-	-	-	-	-
TURB-CNTRL vs. TURB-F	-	-	-	*	**
TURB-CNTRL vs. TURB-IF	-	-	-	-	-
TURB-I vs. TURB-F	-	-	-	-	-
TURB-I vs. TURB-IF	-	-	-	-	-
TURB-F vs. TURB-IF	-	-	-	-	-

The predator treatments were: CNTRL  =  control, no added predators, I =  invertebrate predators (*Chaoborus flavicans*), F =  fish, and IF  =  both invertebrates and fish as predators. (** p<0.01, * p<0.05, - no significant difference).

## Discussion

### Effects of turbulence alone

Except for *P. pediculus*, turbulence alone did not affect the zooplankton, nor did it affect the average size of any of the taxa studied. The between-treatment differences in the values of bottom-up forces such as temperature and phytoplankton availability were negligible. Thus, the between-treatment differences observed in the zooplankton were presumably caused by predation. This was supported by the equal abundance of cladoceran embryos and copepod nauplii in the control treatments with and without turbulence. The non-consumptive effects of predators, reported e.g. by Heuschele et al. [65] were not studied in this paper. However, no effect of various predator treatments on the abundances of either cladoceran embryos and copepod nauplii, or their ratios to adult crustaceans was detected, suggesting that predators did not substantially influence the crustacean reproduction during the study period.

The absence of turbulence effects confirms that the function of the pumps used to generate turbulence did not affect the zooplankton. The decrease in *P. pediculus* under turbulent conditions was expected, because it is a species inhabiting sheltered stagnant habitats and is very vulnerable to environmental stress [66], [67].

### Effects of turbulence on zooplankton biomass via *Chaoborus* predation

Under calm conditions with chaoborids, no significant decrease in crustacean biomass was detected. Turbulence, however, adduced the top-down control exerted by *C. flavicans* on zooplankton, supporting the view that small-scale turbulence enhances the feeding rate of tactile invertebrate predators [9], [28]. The minor effect of *Chaoborus* predation on zooplankton under calm conditions confirms the hypothesis that, at intermediate *Chaoborus* densities other, e.g. physical factors may strongly influence the amplitude of predation on zooplankton. The present results suggest that, turbulence can be considered one of such factors behind the reported, divergent effects of intermediate *Chaoborus* densities on zooplankton [54], [55].

An increase in *RMS* velocity from 0.3 cm s^−1^ in the calm treatments to 1.4 cm s^−1^ in the turbulent treatments should theoretically cause a fourfold increase in the contact rate of *C*. *flavicans* with their prey [9], [68]. Under low visibility conditions, on the other hand, reduced reactive distances [69], [70] can directly affect the estimated numbers of prey entering the capture volume. However, tactile ambush predators, such as *Chaoborus* larvae, do not rely on vision when hunting, and are independent on the visibility conditions. Yet, they are largely limited to attacks on prey entering their effective strike area [71] and thus essentially depend on the movement of prey. Consequently, elevated levels of turbulence can enhance the numbers of encountered prey, thus also affecting the capture efficiency. Since the size distribution of zooplankton with small average individual size was suitable for *C*. *flavicans* larvae [57], the increase in contact rate was reflected in a considerably elevated crustacean prey consumption rate. Under turbulence, cladocerans, especially bosminids, were depressed by chaoborids, which is in accordance with the feeding habits of *C. flavicans* larvae. Instar III and IV larvae of *C. flavicans* usually show a strong positive selection for bosminids [52], [57], [72]. Moreover, increased consumption of crustaceans under turbulence probably released rotifers from the predation pressure of invertebrate predators. The proportion of rotifers decreased in CALM-CNTRL, TURB-CNTRL, and CALM-I and increased in all others. At the same time, the proportion of rotifers increased in all those treatments, in which *P*. *pediculus* collapsed. *Polyphemus pediculus* is predaceous and feeds mainly on rotifers [67]. Thus, our results suggest that predation losses of rotifers to invertebrates may decrease due to turbulence, both because of alterations in the selective feeding and survival of invertebrate predators. The seasonal decrease in rotifer biomass in all the treatments during the experiment, on the other hand, was due to natural summertime succession. In the forest lakes of the Evo area, the abundance of rotifers often decreases steeply from July to August [73]. The predominance of cladocerans over copepods and the occurrence of *B*. *longirostris* as the most abundant crustacean species are also common phenomena in humic forest lakes in Finland [74], [75].

The presence of *C. flavicans* predation favored the larger size classes of zooplankton both with, and without turbulence. This was expected, because *Chaoborus* larvae are gape-limited predators that feed mainly on small prey species, releasing large-bodied species from predation pressure, often resulting in their predominance [15], [76]. Most of the zooplankton prey items found in the mesocosms were in a size range (<1700 µm total length), enabling *Chaoborus'* ingestion [77]. Under calm conditions, chaoborids mainly selected crustaceans <500 µm in length resulting in the predominance of larger individuals such as *P. pediculus*. Under turbulent conditions, however, *P. pediculus* was largely suppressed by turbulence alone, in addition to predation exerted by chaoborids, which led to a less pronounced development in the size-class distribution. Yet, the size selectivity of *Chaoborus* predation was probably not affected by turbulence, since larger crustaceans also tended to predominate towards the end of the study period in TURB-I. Turbulence could affect the size selectivity of predators by affecting pursuit, which is the most vulnerable postencounter process [78]. However, in the case of chaoborids the pursuit time is very short and thus insensitive to turbulence [24], [79].

### Effects of turbulence on zooplankton via fish predation

Under calm conditions, fish had a much stronger effect on cladocerans than did chaoborids. Bosminids and *P. pediculus* especially were depressed by fish. This was expected, because these were the most abundant cladocerans, and cladocerans are preferred over copepods by planktivorous perch and roach [80], [81], [82]. Accordingly, in calm water with fish the biomass of cyclopoids was 2.5 times higher than in calm water without predators. This was explained by reduction in the biomass of the predaceous cladoceran *P*. *pediculus*, which led to increased rotifer biomass, consequently increasing the food available for cyclopoid copepods.

The TURB-F treatment revealed unexpected phenomena. Here, the fish-induced reduction in cladoceran biomass was slightly smaller under turbulent than under calm conditions. The biomass of cyclopoids, however, which increased steeply in calm water with fish, was considerably reduced in turbulence. While both *P*. *pediculus* and cyclopoid copepods were reduced in the TURB-F treatments, at the same time the biomass of rotifers was enhanced. The observation corroborated the assumption that the reduction in *P*. *pediculus* in CALM-F led to increased cyclopoid biomass, due to the increased amount of available food items.

This finding challenges the previous view that small-scale turbulence does not affect the feeding of juvenile or adult fish, due to their high swimming speed [22]. On the other hand, the result was in concordance with Pekcan-Hekim et al. [25], who found a significant interaction effect between inorganic turbidity and turbulence on the feeding efficiency of planktivorous perch. In clear water, turbulence does not affect fish feeding, since their lengthy reactive distances together with high maneuverability enable them to successfully prepare an attack and catch the prey, regardless of the turbulence level. Under low-visibility conditions, the reactive distance is lowered and turbulence brings more prey items into the reactive volume of fish, compensating for the time lost in searching for prey [25].

The differential effect of fish on cladocerans and copepods in calm and turbulent water indicated that in highly colored water turbulence affects the selectivity of fish predation. Planktivorous fish are size-selective feeders and their predation, in contrast to the predation by invertebrates, usually leads to the dominance of small zooplankton species, resulting in the decreased proportion of large cladocerans and increased proportion of cyclopoid copepods [12], [83]. This also holds for perch and roach [84]. Accordingly, the large and conspicuous *P*. *pediculus* was eradicated from all treatments in which fish were present, regardless of turbulence. At low light intensities, planktivorous fish may lose their capability for size-selective feeding [85]. However, our results indicated that fish were able to feed on larger zooplankton, despite the low-visibility conditions, since the larger size classes were reduced by fish both with and without turbulence. No significant differences between the CALM and TURB treatments with fish as predators were found, suggesting that turbulence did not directly affect the size-selectivity of fish, in contrast to calm conditions. Dower et al. [23] found that larval fish selected on average larger zooplankters under turbulent than under calm conditions. This was explained by the fact that fish showed a longer reaction distance for larger prey. Hence, under turbulent conditions a longer time was required for the large prey to pass through the perceptive volume of the fish, thus making them more vulnerable to predation. Our experiments showed that cyclopoid copepods especially were suppressed by fish under turbulent conditions, suggesting that fish change their selectivity from cladocerans towards copepods when turbulence is introduced into a dark-water system. Similar results were obtained from another experiment focusing on the effects of water quality and turbulence on food selection; fish preferred copepods in highly colored water when turbulence was present (Z. Pekcan-Hekim, unpublished data). This was due to the associations between prey-dependent behavior and changes in reaction distances. Saiz and Alcaraz [86] showed that an *?* value of the order of 10^−6^ m^2^ s^−3^ may affect the swimming habits of copepods, thus making them more vulnerable to fish predation, due to decreased escape ability and, on the other hand, increased conspicuousness. In the present study, cyclopoid copepods were nearly twice the size of bosminids (preferred by fish in addition to *P*. *pediculus* under calm conditions). Our results thus suggest that turbulence can affect the size-selective feeding of fish by changing the preferred food item to a larger species.

### Effect of turbulence on interactive predation by invertebrates and fish

Very few differences in zooplankton between fish predation and combined fish-*Chaoborus* predation were observed. This suggests that the addition of chaoborids to the F treatments did not result in additional effects on zooplankton. Under calm conditions this could be expected, since chaoborids only weakly affected the zooplankton biomass when turbulence was not present. In turbulence, however, the addition of *Chaoborus* predation to fish predation could have affected the zooplankton, because in turbulence chaoborids alone had a strong effect. The weak impact of chaoborids in TURB-IF thus indicated that fish reduced their feeding rate. This could have happened through reduction of the *Chaoborus* density via fish predation or through changes in the behavior of chaoborids.

Co-occurrence of planktivorous fish and *Chaoborus* larvae often leads to intraguild predation, in which the intraguild prey (*Chaoborus*) is preyed upon by fish [87]. Perch and roach feed intensively on *C. flavicans* larvae when they are available [88], [89] and turbulence can enhance the prey capture success of perch feeding on chaoborids [25]. Turbulence could thus turn fish predation pressure from zooplankton to chaoborids. The results revealed that this probably happened; intraguild predation occurred in the TURB-IF treatment especially. Increasing consumption of large invertebrate prey by fish can reduce the predation pressure that fish exert on herbivorous zooplankton [90], [91]. Accordingly, in TURB-IF the biomass of cyclopoids was significantly higher than in TURB-F. Cyclopoids were the main prey of fish in turbulent water, and the availability of chaoborids in TURB-IF clearly decreased fish predation on them. This does not, however, exclude the possibility that fish also affected the behavior of chaoborids. When chaoborids cannot avoid predation by occupying low-light and low-oxygen refuges in deep water, they often burrow into the bottom [92], [93]. Such predator-avoidance behavior would also reduce the feeding rate of chaoborids. Decreased food consumption is a commonly reported consequence of refuge use for prey animals [94], [95].

### Conclusions

Climate models predict increasing wind and storm activities [96], resulting in increasing turbulent velocities, especially within the mixed surface layer of lakes. Wind-driven stress is one of the main forces generating turbulence in aquatic ecosystems [97]. Additionally, especially in small, sheltered lakes, convection can be a larger mixed-layer turbulence source than wind shear [98], since it originates from the diurnal warming and cooling of surface water masses and does not require wind as an energy source.

The shallow ponds used in the present study represented a situation, in which turbulence is nearly spatially homogeneous. In natural water bodies, on the other hand, e.g. wind-shear causes vertical variation in turbulence levels [99]. Climatic changes might thus also move the turbulent regions of natural water bodies vertically. In such lakes, in which variations in turbulence climate would extend to water layers where predation occurs, turbulence-induced changes in the feeding efficiency of various predators may have considerable effects on lower trophic levels. Our present results supported the hypothesis that in brown-water lakes, dominated by tactile invertebrate predators, a moderate increase in turbulence can substantially influence zooplankton via effects on predation pressure. The effect was strongest for cladocerans, which is noteworthy, since in lakes cladocerans are the most important consumers of phytoplankton. Thus, in invertebrate-dominated lakes, increases in turbulence and especially contemporaneous increases in water color and turbulence are likely to cascade to primary producers [16]. In a fish-dominated system, turbulence increased predation on copepods, while predation on cladocerans was decreased. In a system inhabited by both invertebrates and fish as predators, the effect of both was reduced, due to intraguild predation. In numerous lakes, however, due to the refuges provided by low-oxygen layers, invertebrates can be the main predators of zooplankton despite the presence of fish [52]. In such lakes, intermediate turbulence may strongly affect the top-down control of zooplankton via enhancement of invertebrate predation and may possibly even turn the dominance from fish to invertebrates.

Decreasing visibility conditions and, on the other hand, increasing turbulence conditions, are both predictable changes in the abiotic environment of lakes [33], [34], [38]. Our novel findings suggest that, depending on the dominating planktivores, the forthcoming changes in abiotic factors can have significant consequences for lower trophic levels, with possible implications even for cascading trophic interactions.
